# Enhancing emergency department charting: Using Generative Pre‐trained Transformer‐4 (GPT‐4) to identify laceration repairs

**DOI:** 10.1111/acem.14995

**Published:** 2024-07-31

**Authors:** Jaskaran (Karan) Bains, Christopher Y. K. Williams, Drake Johnson, Hope Schwartz, Naina Sabbineni, Atul J. Butte, Aaron E. Kornblith

**Affiliations:** ^1^ Department of Emergency Medicine University of California, San Francisco San Francisco California USA; ^2^ Bakar Computational Health Sciences Institute University of California, San Francisco San Francisco California USA

Large language models (LLMs), such as OpenAI's Generative Pre‐Trained Transformer‐4 (GPT‐4), can generate, audit, and process data without domain‐specific training. LLMs have many potential health care applications but require validation and testing before deployment. Much of the current LLM research in health care has focused on supporting clinical decision making.^1^ Charting augmentation is another potential area for LLM application, with a lower risk of patient harm than applications that directly influence medical decision making.

In particular, procedure documentation may be a simple but high‐impact charting use case for LLMs. In settings like the emergency department (ED), where procedures are a routine part of clinical practice, the time‐consuming task of completing procedure notes is often neglected. This oversight can have serious implications for patient care, data integrity, and the financial viability of healthcare organizations. Many hospitals employ operations and billing specialists who manually review charts to identify missed procedure documentation, an expensive, labor‐intensive process that remains error‐prone.^2^ The retrospective nature of procedure documentation exacerbates clinician burnout by increasing postshift documentation burden.^3^

This procedure documentation workflow—simple relative to other charting tasks, monotonous for clinicians, and financially important with minimal patient risk—provides an ideal use case for LLMs. However, an essential first step prior to LLM integration is determining whether the technology can accurately identify patient encounters requiring procedure documentation. Our study focused on laceration repairs, a common procedure accounting for more than 8% of ED visits.^4^ We evaluated GPT‐4 performance in identifying patient encounters requiring laceration repair procedure documentation.

We performed a cross‐sectional study of the publicly available Medical Information Mart for Intensive Care (MIMIC)‐IV‐Note 2.2 database. MIMIC‐IV contains deidentified hospital data spanning 2008–2019 from Beth Israel Deaconess Medical Center, with prior institutional review board approval (see also Supplemental Methods).^5^

Our initial data set consisted of all MIMIC‐IV discharge summaries which contained the keyword “laceration” (case‐insensitive), including both elective surgical admissions and patients admitted from the ED. We focused on admitted patients to increase the cohort complexity for GPT‐4 analysis. We included a small number of elective surgical admissions to evaluate GPT‐4 performance in identifying lacerations repaired in the operating room, which do not require a separate procedure note. Discharge summaries for ED admissions included the initial history and physical examination for the patient. Three trained human reviewers (JB, HS, NS) reviewed a random sample of these encounters to create human labels for patient charts requiring laceration repair documentation according to prespecified criteria (Table S1). Ten percent of encounters were independently labeled by two reviewers to determine inter‐rater reliability using Cohen's kappa.

We randomly divided the labeled data set into (1) a development set for prompt engineering and (2) an independent test set (Figure S1). Encounters with discharge summaries exceeding the ~8000 token (approximately 6000 word) GPT‐4 context window were excluded, as this was the largest GPT‐4 context window available to us at the time of the study. We prompted GPT‐4 to review each discharge summary and determine whether a laceration repair procedure note was required. We evaluated GPT‐4 performance against the human labels using the following metrics: sensitivity, specificity, positive and negative predictive values and likelihood ratios, accuracy, and F1 score. To better understand reasons for GPT‐4 mislabeling, we subsequently conducted an unblinded, post hoc re‐review of the following encounters: (1) all encounters in which GPT‐4 and human labels were discordant, (2) all encounters with concordant labels requiring laceration repair documentation, and (3) a random sample of encounters with concordant labels not identifying laceration repairs.

We reviewed 800 MIMIC‐IV discharge summaries for inpatient encounters. These encounters were randomly divided into a development set of 50 and a test set of 732 encounters. Eighteen encounters exceeded the token count and were excluded. Twenty of these encounters (2.7%) were elective surgical admissions, and 97.3% were admissions from the ED. In total, 163 of 732 (22.3%) encounters required a laceration repair procedure note as determined by human review. The mean age of the cohort was 57 years, and 22 patients (3%) died while hospitalized. Other demographic information is reported in Table S2. Cohen's kappa was 0.822 for the 80 encounters labeled by two reviewers.

GPT‐4 performance on the test set is reported in Table 1. Sensitivity was 77.3% and specificity was 94.6%, with overall accuracy of 90.7% and F1 score of 0.788. These results were unchanged when elective surgical admissions were excluded.

**TABLE 1:** Test characteristics of GPT‐4 in identifying ED patient encounters requiring laceration repair procedure notes (*n* = 732).

		Human review	
		+	−
GPT‐4 review	+	126	31
	−	37	538
Test set *n*		732	
F1 score		0.788	
Accuracy		90.7%	
Sensitivity		77.3%	
Specificity		94.6%	
PPV		80.3%	
NPV		93.6%	
LR+		14.188	
LR−		0.240	

Abbreviations: GPT‐4, Generative Pre‐trained Transformer‐4; LR, likelihood ratio; NPV, negative predictive value; PPV, positive predictive value.

There were 31 encounters that GPT‐4 identified as requiring a laceration repair note when human reviewers did not. On re‐review, seven of these encounters had erroneous human labels. Of the remaining 24 encounters mislabeled by GPT‐4, 12 included lacerations repaired at another hospital before transfer or during a prior ED visit.

Thirty‐seven encounters identified by GPT‐4 as not requiring a laceration repair note were discordant with human review. On manual re‐review, 23 of these encounters were mislabeled during the original human review: seven with laceration repair at another hospital, 13 with a laceration identified but no specific repair mentioned, and three with a repaired laceration in the initial physical examination. The latter two categories met predefined exclusion criteria (Table S1), yet human reviewers determined that they required documentation based on clinical context not accounted for in the labeling instructions.

In total, the most common clinical scenarios among discordant labels were lacerations repaired at another hospital or a prior visit (22/68, 32%), lacerations identified with no specific repair mentioned (20/68, 29%), and those repaired by consulting surgical services (18/68, 26%).

Manual re‐review of all 126 patient encounters with concordant labels requiring a laceration repair note revealed that initial human review had an accuracy of 90% (113/126). Twelve of the 13 inaccuracies on initial human review were due to lacerations repaired at another hospital or ED visit. Manual re‐review of a random sample of 126 concordantly labeled encounters that did not require laceration repair documentation demonstrated 100% accuracy.

GPT‐4 accurately identified patient encounters requiring a laceration repair note using a patient's discharge summary, with an F1 score of 0.788 and accuracy exceeding 90%. Our results underscore the potential application of LLMs to identify patient encounters requiring procedure notes, an important first step to improving ED procedure documentation. To our knowledge, no study to date has assessed GPT‐4's performance on such a task, limiting direct comparisons of our findings. However, our results compare favorably to GPT‐4 accuracy in other charting domains, which has ranged widely from 25% when selecting CPT codes for spinal procedures to 89% when identifying high‐acuity ED patients.^1^, ^6^

Identification of encounters requiring procedure documentation is the most difficult task in the procedure documentation workflow. Once relevant encounters are identified, the assessment of whether procedure documentation was “missed” simply requires querying whether a procedure note is present. After missed procedures were identified, even simple interventions such as pages and email reminders to clinicians have successfully increased documentation rates.^7^

Although GPT‐4 achieved relatively high accuracy in this study, we do not consider its current performance sufficient for clinical deployment. With current sensitivity and specificity, for every 1000 patients, GPT‐4 would miss 24 encounters deemed by humans to require laceration repair notes while sending 48 inappropriate procedure note completion alerts to clinicians. Although our specificity of 95% compares favorably to other clinical GPT‐4 tasks,^8^ this number of discordant labels nevertheless risks exacerbating alert fatigue. Future studies should consider the addition of lower acuity ED patients, more heterogeneous selection of electronic health record data elements for identifying procedures, routing notes to the appropriate clinician, and the effects of prompt engineering on model calibration to reduce the rates of discordant labels.

GPT‐4's true performance may have exceeded these metrics, as many of the discordant labels were the result of scenarios not precisely addressed in labeling criteria, alongside errors in initial human review. Concordant labels had very low human error rates, possibly reflecting lower complexity scenarios. GPT‐3.5‐turbo has previously demonstrated higher accuracy in text annotation than untrained human annotators, as well as higher inter‐rater reliability than both untrained and trained annotators.^9^ The discrepancies between GPT‐4 and our trained annotators, who in some cases used clinical intuition not present in the labeling criteria, highlight the importance of re‐reviewing discordant encounters to inform future LLM prompting. Although our labeling criteria addressed scenarios such as transfers from another hospital, initial physical examinations with repaired lacerations, bedside repair by consulting services, and encounters in which a laceration was identified but no repair was explicitly mentioned, these scenarios were nevertheless sources of discordance between human and GPT‐4 review. A focus on even more specific prompting in these areas could improve future performance.

Our study has several other limitations. First, the MIMIC‐IV data set used was limited to hospitalized patients, with a 3% in‐hospital mortality rate similar to that of national ED admissions (2.7%).^10^ This artificially raised the complexity of our cohort and limits generalization of our results to a representative ED population that includes discharges. Second, the enrichment of the test data using a keyword search may not reflect real‐world populations and contributes to overrepresentation of lacerations (22% prevalence in our study compared to the 8% seen in national ED data^4^) and false positives. Third, the unblinded post hoc re‐review could introduce bias. Future research should validate our findings across additional procedures with a complete medical record, which could improve GPT‐4 performance.

As each iteration of GPT has consistently outperformed prior versions on health care tasks,^6^, ^8^ we expect future LLMs to further improve test characteristics. Testing current models and developing applications for procedure documentation holds value even without immediate clinical use. As improved LLMs are released, health care organizations ready to integrate them will gain an edge in achieving real‐time identification of missed procedure documentation in the ED and other clinical settings.

## AUTHOR CONTRIBUTIONS

Jaskaran (Karan) Bains and Aaron E. Kornblith conceived the study. Jaskaran (Karan) Bains, Aaron E. Kornblith, Christopher Y.K. Williams, and Drake Johnson designed the methods. Jaskaran (Karan) Bains, Hope Schwartz, and Naina Sabbineni manually reviewed patient encounter data for labeling. Drake Johnson managed the data, including quality control. Christopher Y.K. Williams performed data analysis, sharing data with the GPT‐4 model using UCSF Versa, a secure, HIPAA‐compliant interface. Jaskaran (Karan) Bains drafted the manuscript, and all authors contributed substantially to its revision. Jaskaran (Karan) Bains and Aaron E. Kornblith take responsibility for the paper as a whole.

## FUNDING INFORMATION

AK is funded by the Eunice Kennedy Shriver National Institute of Child Health and Human Development (1K23HD110716‐01).

## CONFLICT OF INTEREST STATEMENT

AK is a co‐founder and consultant to CaptureDx. This entity did not have any role in the design, planning, or execution of the study or interpretation of the findings. AB is a co‐founder and consultant to Personalis and NuMedii; consultant or advisor to NIH, JAMA, Mango Tree Corporation, Samsung, Geisinger Health, Washington University in Saint Louis, University of Utah, and in the recent past, 10× Genomics, Helix, Pathway Genomics, and Verinata (Illumina); has served on paid advisory panels or boards for Regenstrief Institute, Gerson Lehman Group, AlphaSights, Covance, Novartis, Genentech, Merck, and Roche; is a shareholder in Personalis and NuMedii; is a minor shareholder in Apple, Meta (Facebook), Alphabet (Google), Microsoft, Amazon, NVIDIA, AMD, Snap, 10x Genomics, Doximity, Regeneron, Sanofi, Pfizer, Royalty Pharma, Moderna, BioNtech, Invitae, Pacific Biosciences, Editas Medicine, Eli Lilly, Nuna Health, Assay Depot (Scientist.com), Vet24seven, Snowflake, Sophia Genetics, and several other non–health‐related companies and mutual funds; and has received honoraria and travel reimbursement for invited talks from Johnson and Johnson, Roche, Genentech, Pfizer, Merck, Lilly, Takeda, Varian, Mars, Siemens, Optum, Abbott, Celgene, AstraZeneca, AbbVie, Westat, Applied Research Works, Acentrus, ALDA, and many academic institutions, medical‐ or disease‐specific foundations and associations, and health systems. Atul Butte receives royalty payments through Stanford University, for several patents and other disclosures licensed to NuMedii and Personalis. Atul Butte's research has been funded by NIH, FDA, Peraton (as the prime on an NIH contract), Priscilla Chan and Mark Zuckerberg, the Barbara and Gerson Bakar Foundation, Genentech, Johnson and Johnson, Chan Zuckerberg Science, Robert Wood Johnson Foundation, Leon Lowenstein Foundation, Intervalien Foundation, and in the past, the March of Dimes, Juvenile Diabetes Research Foundation, California Governor's Office of Planning and Research, California Institute for Regenerative Medicine, L'Oreal, and Progenity. None of these entities had any role in the design, planning, or execution of the study, or interpretation of the findings. The other authors declare no conflicts of interest.

## Supporting information


Data S1:
