# Supplementary material for: Enhancing emergency department charting: Using Generative Pre‐trained Transformer‐4 (GPT‐4) to identify laceration repairs
Source: Acad Emerg Med. 2024 Jul 31;32(1):94–7. doi: 10.1111/acem.14995 (PMC11726143; doi:10.1111/acem.14995)
Supplement: Supplementary file 1 — Data S1: [file ACEM-32-94-s001.docx]

**Supplemental Material**

**Supplemental Methods**

This study followed the Strengthening the Reporting of Observational Studies in Epidemiology (STROBE) reporting guideline for observational studies. The study was exempt from approval by our Institutional Review Board (IRB) because it used MIMIC-IV-Note (version 2.2), a public, deidentified database of hospital records from Beth Israel Deaconess Medical Center with prior IRB approval.^1^ The authors passed the relevant training course for accessing and extracting the database and obtained the relevant certificate. Sharing of MIMIC-IV data with the GPT-4 model was performed using the secure, HIPAA-compliant Microsoft Azure API interface (‘UCSF Versa’) in accordance with the ‘Responsible use of MIMIC data with online services like GPT’ guidance associated with the MIMIC datasets.^2^ MIMIC-IV contains both discharge summaries for ED and hospital encounters as well as a database of radiology reports. We used only hospital discharge summaries for admitted ED patients (97%) and elective surgical admissions (3%) by using the “discharge” table (radiology, radiology_detail, and discharge_detail tables were not used). The discharge table of MIMIC-IV does not include information on procedure documentation. Additional information about the MIMIC-IV database can be found in the Supplemental References.^3,4^

GPT-4 temperature was set to 0, with the remainder of the settings unchanged from default.

A flowchart of our sample selection is shown in **Supplemental Figure 1.** The pre-specified criteria used by our three independent human reviewers to determine whether patients required laceration repair procedure notes are displayed in **Supplemental Table 1**.

**Supplemental Figure 1: Flowchart of discharge summary selection from MIMIC-IV**

**
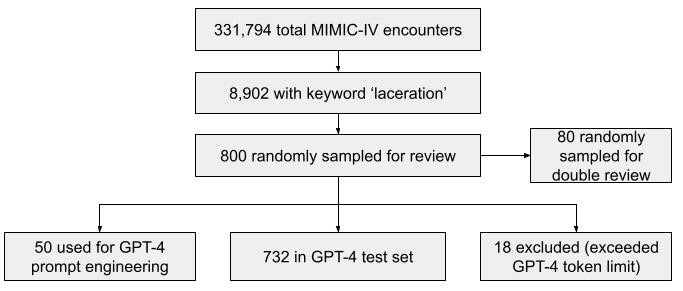
**

**Supplemental Table 1: Labeling Criteria for Human Reviewers**

| **Inclusion Criteria** | **Exclusion Criteria** |
| --- | --- |
| (1) If a patient received a laceration during the current hospital stay that was not done in the operating room (OR)   - Counts even if done by a team other than the emergency room (ER) team - Lacerations can be repaired by sutures (stitches), staples, glue, or even tying hair together in some cases; all of these count as procedures and need a procedure note   (2) If a patient is admitted to the hospital, a laceration repair is mentioned during the hospital stay, and there is no mention of a surgery or the OR, assume the laceration was repaired out of the OR and requires a procedure note  (3) Search terms in addition to “laceration” that can be helpful if you are unsure: stitch, suture, staple, glue, repair | (1) No mention of laceration  (2) Laceration repair done in operating room (OR)  (3) History of laceration in the past but no laceration during this ER/hospital visit  (4) Laceration repaired at another hospital before transfer to this hospital   - If the initial physical exam shows a repaired laceration, then consider it done prior to this visit - If discharge instructions state that laceration was repaired in this visit, consider a procedure note   (5) Lacerations noted to be more than 1-2 days old are very unlikely to be repaired – no note  (6) Any lacerations of internal organs or muscles/tendons are likely to be repaired in operating room and should NOT receive a note – examples (not exhaustive) include: lung, liver, spleen, pancreas, tendon, open fractures  (7) Lacerations sustained during delivery/childbirth are part of the delivery billing process – no note  (8) If there is a small laceration mentioned and no other indication that the laceration was repaired – no note (err on conservative side) |

Prompt engineering was performed on the development set of 50 patient discharge summaries, incorporating the pre-specified inclusion/exclusion criteria provided to human reviewers for labeling (**Supplemental Table 1**). Below is the final prompt used on the test set of 732 discharge summaries:

*“You are an emergency medicine physician seeking to identify whether a patient requires a laceration repair procedure note. Patients require a laceration repair procedure note according to the following criteria:*

*General tips:*

*The most useful sections to examine are generally the “History of Present Illness”, “Physical Exam” and “Brief Hospital Course” sections.*

*The first instance of the word “laceration” has been placed at the top of each note along with the text around it, for easier reference.*

*Patient requires a laceration repair procedure note (INCLUSION CRITERIA):*

*If a patient received a laceration repair during the current hospital stay that was not done in the operating room.*

*Counts even if done by a team other than the emergency room team.*

*Lacerations can be repaired by sutures (stitches), staples, glue, or even tying hair together in some cases - all of these count as procedures and need a laceration repair procedure note.*

*If a patient is admitted to the hospital, a laceration repair is mentioned during the hospital stay, and there is no mention of a surgery or the operating room, assume the laceration was repaired outside of the operating room and requires a laceration repair procedure note.*

*Patient does not require a laceration repair procedure note (EXCLUSION CRITERIA):*

*No mention of a laceration repair.*

*Laceration repair was performed in an operating room.*

*Patient has a history of laceration repair in the past to no laceration repair during this emergency room/hospital visit.*

*Laceration was repaired at another hospital before transfer to this hospital.*

*Tip - if the initial physical exam shows a repaired laceration, then consider the laceration repaired prior to this visit - therefore, no laceration repair procedure note is required.*

*Tip - if discharge instructions state that a laceration was repaired in this visit, consider a laceration repair procedure note.*

*Lacerations noted to be more than 1-2 days old are very unlikely to be repaired - therefore, no laceration repair procedure note is required.*

*Any lacerations of internal organs or muscles/tendons are likely to be repaired in the operating room and should NOT receive a note - examples (not exhaustive) include:*

*Lung laceration, liver laceration, spleen/splenic laceration, pancreatic laceration, tendon laceration*

*Lacerations associated with a fracture (open fracture) are also repaired in the operating room*

*Lacerations sustained during delivery/childbirth are part of the delivery billing process - therefore, no laceration repair procedure note is required.*

*If there is a small laceration mentioned, but there is no other indication that the laceration was repaired - no laceration procedure note is required.*

*Review the below note to determine whether the patient requires a laceration repair procedure note.”*

**Supplemental Results**

Our test sample of 732 patients had a mean age of 57 years. Patients presented to the ED with diverse mechanisms of injury, including high-force blunt and penetrating trauma, but also falls, seizures, and psychiatric complaints. All patients were admitted to the hospital, and either passed away during their hospital course (22/732, or 3.0%) or were discharged to home independently (39%), to a skilled nursing facility or rehab (23.1%), or with home health care (16%), as shown in Supplemental Table 2.

Twenty-two of these patients passed away while in the hospital (3%). The clinical scenarios for each of the 22 patients who passed away during their hospital admission included trauma with intracranial hemorrhage (6), fall with cardiac arrest (5), fall (4), stroke (2), and other presentations including a motorcycle collision. GPT-4 and human labelers agreed on 18/22 (82%) of these patients. Of the four patients in which GPT-4 and human labelers were discordant, two were erroneously labeled by human reviewers on post-hoc review. GPT-4 mislabeled two patients, both of which involved hospital transfers and one of which involved two lacerations, (one repaired at an outside hospital and another which was not).

**Supplemental Table 2: MIMIC Cohort Demographics**

| **Category** | **Count** | **% of Total** |
| --- | --- | --- |
| *Age* |  |  |
| 0-9 | 0 | 0% |
| 10-19 | 13 | 2% |
| 20-29 | 100 | 14% |
| 30-39 | 66 | 9% |
| 40-49 | 92 | 13% |
| 50-59 | 124 | 17% |
| 60-69 | 102 | 14% |
| 70-79 | 96 | 13% |
| 80-89 | 108 | 15% |
| 90+ | 31 | 4% |
| *Gender** |  |  |
| Male | 435 | 59% |
| Female | 297 | 41% |
| *Race/Ethnicity* |  |  |
| White | 508 | 69% |
| Unknown | 75 | 10% |
| Black | 62 | 8% |
| Hispanic | 42 | 6% |
| Other | 22 | 3% |
| Asian | 17 | 2% |
| Other | 6 | 1% |
| *Insurance Status* |  |  |
| Medicare | 281 | 38% |
| Medicaid | 63 | 9% |
| Other | 388 | 53% |
| *Discharge Destination* |  |  |
| Home (independent) | 286 | 39% |
| Skilled nursing facility/rehab | 169 | 23% |
| Home + home health | 117 | 16% |
| Unknown | 89 | 12% |
| In-hospital death | 22 | 3% |
| Other | 49 | 7% |
| *No non-binary/other category provided in source data | | |

**References**

1. Johnson, A., Pollard, T., Horng, S., Celi, L. A., & Mark, R. (2023). MIMIC-IV-Note: Deidentified free-text clinical notes (version 2.2). PhysioNet. https://doi.org/10.13026/1n74-ne17.
2. Responsible use of MIMIC data with online services like GPT. Accessed December 27, 2023. https://physionet.org/news/post/415.
3. Johnson AEW, Bulgarelli L, Shen L, et al. MIMIC-IV, a freely accessible electronic health record dataset. Sci Data. 2023;10(1):1. doi:10.1038/s41597-022-01899-x.
4. Goldberger, A., Amaral, L., Glass, L., Hausdorff, J., Ivanov, P. C., Mark, R., ... & Stanley, H. E. (2000). PhysioBank, PhysioToolkit, and PhysioNet: Components of a new research resource for complex physiologic signals. Circulation [Online]. 101 (23), pp. e215–e220.
